# Measuring the Impact of Health on Economic Growth Using Pooling Data in Regions of Asia: Evidence From a Quantile-On-Quantile Analysis

**DOI:** 10.3389/fpubh.2021.689610

**Published:** 2021-08-31

**Authors:** Cheng-Feng Wu, Tsangyao Chang, Chien-Ming Wang, Tsung-Pao Wu, Meng-Chen Lin, Shian-Chang Huang

**Affiliations:** ^1^School of Business Administration, Hubei University of Economics, Wuhan, China; ^2^School of Business, Wuchang University of Technology, Wuhan, China; ^3^Research Center of Hubei Logistics Development, Hubei University of Economics, Wuhan, China; ^4^Institute for Development of Cross-Strait Small and Medium Enterprise, Wuhan, China; ^5^Department of Finance, Feng Chia University, Taichung, Taiwan; ^6^CTBC Business School, Tainan, Taiwan; ^7^Department of International Business, Ming Chuan University, Taipei, Taiwan; ^8^School of Accounting and Finance, Beijing Institute of Technology, Zhuhai, China; ^9^Department of Business Administration, National Changhua University of Education, Changhua, Taiwan

**Keywords:** quantile-on-quantile, health, economic growth, Asia, pooling data

## Abstract

Health improvement has become a significant social priority since a moderately good human capital condition improves the workforce's abilities, efficiency, and quality of life. A rapid increase in healthcare expenditure is a trend in major developing and developed countries; however, healthcare expenditure widely varies among most Asian countries. Asian countries contribute a significant amount of output to economic development worldwide. The statistical test power is more efficient for the pooling of national data than individual national data because of the economic value and trade integration of regional nations. This is the first study that applies the quantile-on-quantile approach to investigate the influence of the quantiles of healthcare on the quantiles of the economy's growth for pooling forty countries in the Asian region. As the quantile of healthcare expenditure increases in the countries, the impact of healthcare expenditure on the economy's growth does not guarantee an increase. The positive and negative effects of healthcare expenditure on developing the economic relationship will repeatedly occur when the quantiles of the economy's growth increase in the countries. One implication is that the governments should account for problems such as corruption, bureaucracy, underinvestment, and inefficiency in health-related resource utilization.

## Introduction

Health improvement has become a significant social priority since a moderately good human capital condition improves the abilities, efficiency, and quality of life of a workforce. Moreover, through its impact on the output in production and service, human capital accumulation is a primary determinant of economic development (1). Accordingly, health is an essential bridge to link human capital accumulation and economic growth.

A country's condition of health impacts economic development in multiple ways. Manufacturing and service are value added by incorporating expert knowledge, capital equipment, technical expertise, and medical science when public health status increases (2, 3). The determinant of the level of expenditure, the social policy environment, and the quality of services is relative to health outcomes (4). Health outcome relies on competitiveness and quality for individuals, since healthy employees have more ability to create innovation (5, 6). On the demand side, people will demand the best health services as they are richer, and they are thus willing to pay a higher price for more quality private healthcare services. Even if expenditure levels are low, health status would be a better outcome for a country where the government executes its social and health policies well (4). The government's health expenditure is one of the essential factors with which to accumulate human capital as health status has been considered one of the unique elements that affect economic growth for a country (7).

Since human capital is a critical indicator in the endogenous growth model that was proposed in the 1990s, health in a country is noticed by researchers and policymakers in academics and in practice. The health-led growth hypothesis is proposed by Mushkin (8) who addressed that healthcare expenditure in a country is important to increase economic growth. An increase in funds, activities, and efforts related to health is expected to increase the wealth of individuals and society in a country. Previous research mainly suggested three outcomes to show the way that health affects growth, i.e., positive relationship (9–16), negative relationship (1, 17, 18), and neutrality (19–21). On the other hand, certain studies analyze the relationship by pool (panel) data, and the results are mixed depending on different groups of economic development (1, 3, 22–29).

According to the geographic position, health and economic development are vital in Asia countries. From the perspective of economic, Asian countries contributed ~60% of the world GDP per capita in 2017. On the other hand, investigating healthcare expenditure is important for the government to improve the health system and health plan of a country, as a rapid increase of healthcare expenditure is a trend in major developing and developed countries (30, 31). According to the global health expenditure database in World Health Organization, healthcare expenditure as a percentage of GDP varied from 1.5 to 11.5 among Asian countries in 2017. In sum, previous studies investigated the impact of health on economic development in traditional time series models. Limited studies examined the relationship in Asian countries using the quantile-on-quantile method.

This study applies the quantile-on-quantile method which combining conventional quantile regression and non-parametric estimation techniques. To the best of our knowledge, this study is the first to investigate the impact of health on economics by applying the quantile-on-quantile method in Asian countries. The quantile-on-quantile analysis is an advanced method that provides more comprehensive information for the influence of quantiles of health on the conditional quantiles of economic growth. In addition, the pooling of national data is adopted because of the value of economic and trade integration of regional nations. The statistical test power is more efficient for the pooling of national data than individual national data (3). The results will give the government more reference to form health systems and policy strategies for Asian countries.

## Data and Method

In this study, pooling data from forty Asian countries is used to investigate the influence of healthcare quantiles on the quantiles of economic growth. The study uses the percentage of healthcare expenditure on GDP as a proxy of healthcare and real GDP per capita as a measure of economic growth by following prior studies regarding the aspect of the health-growth nexus. The collected annual data from 2000 to 2017 are sourced from the World Bank, and each series of real GDP per capita has a processed logarithm. We examine the order of integration of the variables taken into account in the analysis before we move to empirical investigation. The proxy variables are stationary at the level since the null hypothesis with regard to the variables is significantly rejected in the panel unit root test by using the LLC test (32) and Fisher-PP test (33). Thus, the data are used for the following empirical investigation.

Sim and Zhou (34) introduced the quantile-on-quantile framework incorporating the quantile regression element and a non-parametric methodology to identify asymmetric and spatial attributes for a model over time. The quantile-on-quantile approach is applied in this study to measure the effect of the quantiles of healthcare on the quantiles of economic growth using a data pooling of 40 countries in Asia. The non-parametric quantile regression model implemented in the analysis is seen as follows:

$$Y_{t}=\beta^{\theta }\left(X_{t}\right)+\mu_{t}^{\theta }$$

where *Y*_*t*_ represents real GDP per capita of the samples at period t, *X*_*t*_ represents the percentage of healthcare expenditure on GDP of the samples at period t, θ is the θth quantile of the conditional distribution of real GDP per capita, and $\mu_{t}^{\theta }$ is the quantile residual term whose conditional θth quantile is assumed to be zero. β^θ^(·) is unknown in the model since we lack prior knowledge of the connection between healthcare and economic development. In non-parametric quantile-on-quantile estimations, the bandwidth (denoted as a parameter of h) selection is crucial since it governs the estimated coefficients' smoothness. Following the setting in Sim and Zhou (34), this study sets 5% bandwidth of density function (*h* = 0.05) for optimal parameters to solve the minimization problem.

For more detail on the derivation of the mathematical model in time series, refer to Sim and Zhou (34). The quantile-on-quantile regression combinates the advantage of both quantile regression and non-parametric estimation. The approach applied in this study presents more comprehensive knowledge about how various quantiles of healthcare impact quantiles of healthcare influence different quantiles of economic growth in the results.

## Results

### Quantile-on-Quantile Estimates

**Figure 1:**
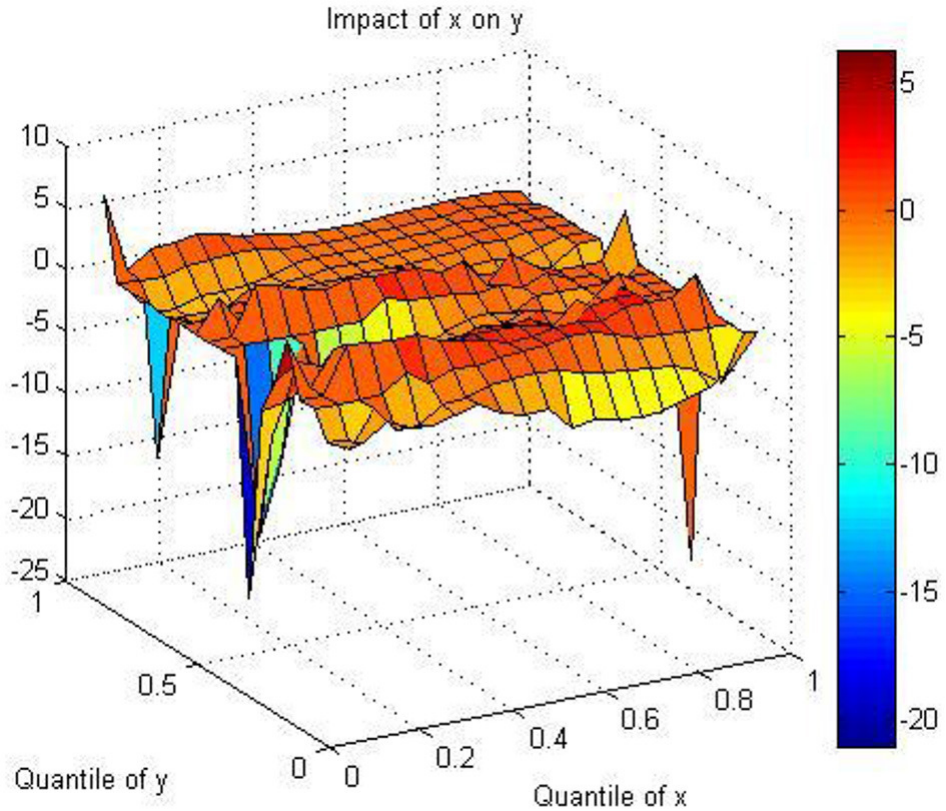
THe estimation of the slope coefficient three-dimensional diagram. X indicates healthcare expenditure; Y indicates economic growth.

This part describes the healthcare and economic growth's empirical results by investigating pooling data by applying the quantile-on-quantile method. Figure 1 illustrates the estimates of the slope coefficient β1(θ, τ), which evaluate the effect of the τth quantile of healthcare expenditure on the θth quantile of economic growth at different values of θ and τ for pooling 40 Asian countries under consideration. For the individual forty countries in Asia, the health-growth nexus is classified into nine groups in a matrix. Based on the evidence in prior studies, the power of the statistical test is more efficient for the national data pooling rather than individual national data. The relationship between healthcare and economic growth is heterogenous for the quantiles of the health-growth nexus.

**Figure 2:**
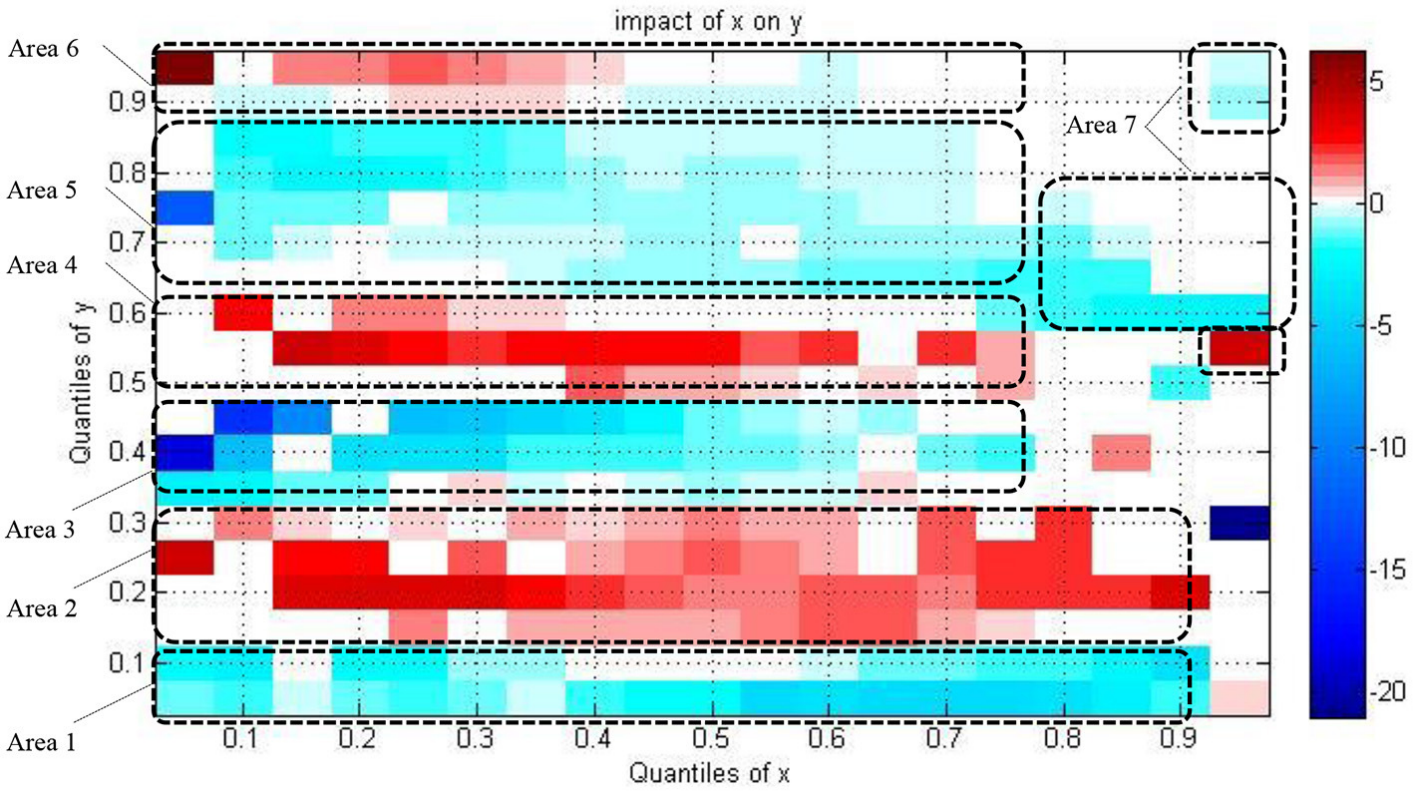
The estimation of the slope coefficient two-dimensional diagram. X indicates healthcare expenditure; Y indicates economic growth.

Figure 2 illustrates the statistically significant coefficient values of the explanatory variable in a two-dimensional diagram to more clearly present main information in the results. The significant coefficient values and the relationship between variables of interest move from low to high as the color bar shifts from blue to red. The colored bar is scaled which shows the numerical values associated with the different colors for the coefficients, following other studies in the literature that use the QQR technique (34–37). This study discusses seven major areas in the results as follows.

In area 1, a negative impact of healthcare on the economy's growth is detected at the lowest to high quantiles of healthcare expenditure (0.05–0.90) with the lower quantiles of economic growth (0.05–0.1) as shown by the blue areas. The negative impact of healthcare on the economy's growth is more substantial at middle and high quantiles of healthcare expenditure rather than low quantiles as the color bar is dark blue at middle and high quantiles of healthcare expenditure.

Focusing on area 2, the relationship positively running from healthcare to economic growth in the countries is located in the point that incorporates the lowest to high quantiles of healthcare expenditure (0.05–0.90) with the low quantiles of economy's growth (0.15–0.3) as shown by the red areas. The positive impact of healthcare on the economy's growth is strong at low quantiles of healthcare expenditure. It then becomes weak at middle quantiles and converts into strong at high quantiles of healthcare expenditure. The color bar is dark red at low quantiles of healthcare expenditure; it becomes light at middle quantiles; it again becomes dark red at high quantiles. The result confirms the evidence of Rana et al. (38) and Chaabouni and Saidi (27) who reported that the relationship runs from healthcare to economic growth in low-income countries, although Sarwar et al. (20) noted that insufficient evidence shows that healthcare leads the economy's growth.

In area 3, the linkage between healthcare and the economy's growth is negative in the countries situated in the zone that combines the lowest to upper-middle quantiles of healthcare expenditure (0.05–0.75) with the lower-middle quantiles of the economy's growth (0.35–0.45) as shown by blue areas. The negative impact of healthcare on the economy's growth is strong at low quantiles of healthcare expenditure, becoming weak at middle quantiles and weakest at upper-middle quantiles of healthcare expenditure. Referring to area 3 in Figure 2, the blue color is relatively dark at low quantiles of healthcare expenditure rather than upper-middle quantiles.

In area 4, a positive relationship running from healthcare to economic growth in the countries exists, a mixing the low to upper-middle quantiles of healthcare expenditure (0.1–0.75) with the middle to upper-middle quantiles of the economy's growth (0.5–0.6) as shown by red areas. The positive impact of healthcare on the economy's growth is strong at low quantiles of healthcare expenditure, then become weak at middle quantiles and become weakest at upper-middle quantiles of healthcare expenditure. The dark red color becomes light as the quantiles of healthcare expenditure increase in this area. Somehow, the nexus is positive at the point, mixing the highest quantiles of healthcare expenditure (0.95) and middle quantiles of economic growth (0.55). This evidence corroborates that of Chaabouni and Saidi (27) who found the causality running from healthcare to the economy's growth in middle-income countries.

Focusing on area 5, the relationship between healthcare and economic growth is negative in the countries located in the region that pair the lowest to high quantiles of healthcare expenditure (0.05–0.85) with the upper-middle to high quantiles of the economy's growth (0.65–0.85) as shown by blue areas. The healthcare expenditure's negative impact on the economy's growth is strong at the lowest healthcare expenditure quantiles. Then, it becomes weak at middle quantiles and becomes strong again at high quantiles of healthcare expenditure. The dark blue color becomes light as the quantiles of healthcare expenditure increase; however, the dark blue color shows up again at high quantiles of healthcare expenditure.

In area 6, the association between healthcare and the economy's growth is positive in the countries located in the zone that pair the lowest to high quantiles of healthcare expenditure (0.05–0.85) with the high to highest quantiles of the economy's growth (0.9–0.95) as shown by red areas. Healthcare expenditure's positive impact on the economy's growth is more substantial at the lowest to middle quantiles of healthcare expenditure rather than high quantiles. The color bar is dark red at low quantiles of healthcare expenditure, and it becomes light at the middle quantiles. Interestingly, in area 7, healthcare's negative impact on the economy's growth is shown for the countries positioning at the upper-middle to highest quantiles of healthcare expenditure (0.8–0.95) and middle to highest quantiles of the economy's growth (0.6–0.95) as shown by blue areas. Healthcare expenditure's negative impact on the economy's growth is stronger at middle and high quantiles of healthcare expenditure rather than low quantiles. Furthermore, the results complement those of Amiri and Ventelou (39) and Rana et al. (38) who find in their study of the health-economic nexus in the OECD countries and 161 countries that health influences economic growth in high-income countries. However, the results differ from those of Sarwar et al. (20) who indicated insufficient evidence of causality from healthcare to economic growth for high-income countries.

In sum, although healthcare spending is on the rise in society, economic development does not ensure growth. Specifically, the relationship between healthcare expenditure and the economy's growth does not exist at upper-middle quantiles of healthcare expenditure. The positive effect of healthcare expenditure on the economy's growth relationship and the negative impact of healthcare expenditure on the economy's growth relationship will repeatedly occur when the economy's growth increases in the countries.

### The validity of the Quantile-on-Quantile approach

**Figure 3:**
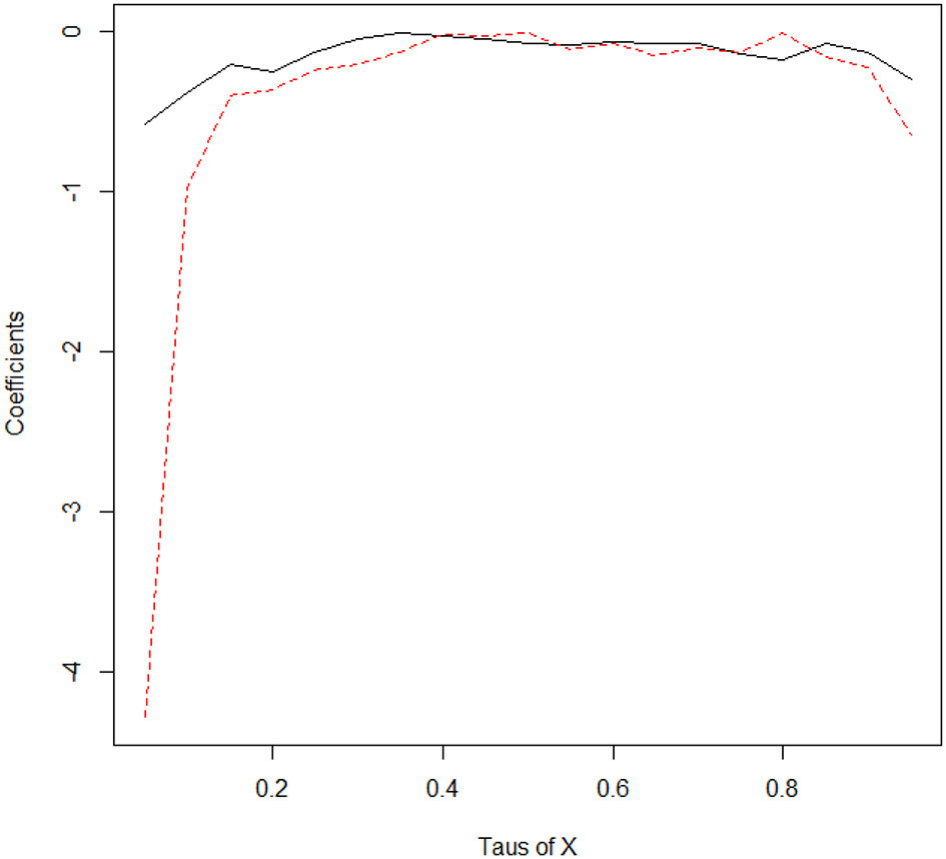
The coefficients in different quantile from the estimation of quantile regression and quantile-on-quantile estimate. The black line indicates the estimates of coefficients in different quantiles for the quantile regression method. On the other hand, the dashed line indicates the coefficients' estimates in different quantiles for the averaged quantile-on-quantile. X indicates healthcare expenditure.

In the current analysis, the quantile-on-quantile method was applied to regress the θth quantile of economic growth on the τth quantile of healthcare expenditure. The average values of slope parameters relating to the quantile-on-quantile regression approach should be approximately similar to those of traditional quantile regression to validate the results of the quantile-on-quantile regression approach in the previous discussion. The quantile regression parameter indexed only by θ can be generated by averaging the quantile-on-quantile regression parameter along τ. The slope coefficient of the quantile regression model can be obtained by the following formula, where this coefficient measures the impact of healthcare expenditure on the distribution of economic growth, which is expressed by γ_1_(θ):

$$\gamma_{1}=\left(\theta \right)\equiv {\bar{\hat{\beta }}}_{1}\left(\theta \right)=\frac{1}{s}\sum_{\tau }{\hat{\beta }}_{1}\left(\theta ,\tau \right)$$

where s is the number of quantiles and τ = [0.05, 0.10, …, 0.90, 0.95]. Figure 3 provides a comparative assessment of the quantile-on-quantile regression approach with the quantile regression method; it confirms the previous findings and follows similar trends.

## Discussion and Conclusion

The prior research provides insufficient information on whether the health-growth nexus differs across countries under or over-spending regarding healthcare. To the best of our knowledge, this is the first study attempting to analyze the true dependency causality running from health to economic growth by considering 40 Asian countries. We used quantile-on-quantile regression techniques to investigate different health quantiles' effects on the entire range of economic growth. In sum, as the healthcare quantiles expenditure increase in the countries, the impact of healthcare expenditure on the economy's growth does not guarantee an increase. On the other hand, the positive effect of healthcare expenditure on the economy's growth relationship and the negative impact of healthcare expenditure on the economy's growth relationship will repeatedly occur when the economy's growth increases in the countries. Specifically, the relationship running from healthcare to the economy's growth is not favorable in the countries where the governments spend high healthcare expenditure.

As healthcare expenditure changes from low to high quantiles, the impact of healthcare expenditure on the economy's growth becomes weak for most quantile of economic growth. The results corroborate with Ye and Zhang's (40) study, which analyzed the effect of health expenditure on the economy's growth in OECD countries. They indicate that technology innovation, to provide value creation, mainly contributes to the economy's growth in developed countries. Thus, the contribution of healthcare expenditure to economic growth is weak. On the other hand, the strong impact in low quantile healthcare expenditure in our result supports the prior study of Rana et al. (38). Rana et al. (38) investigated low-income countries and addressed how the external resources contributed from the foreign countries to low-income countries are the possible reason to justify the impact. Poverty and inequality lead to the spread of infectious diseases that affects public health (41–43). Thus, the governments in low-income countries should construct health systems with sufficient investment and health workers that contribute to the citizens, preventing disease.

The sign of the nexus between healthcare expenditure and economic growth repeatedly changes in terms of the extent of different quantile combinations of health expenditure and economic growth. Grossman (44) presents the concept of “health capital” as part of the demand model for “good health.” Based on the insights, healthcare expenditure in human capital is not guaranteed to satisfy health outcomes. However, healthcare expenditure possibly leads to good health outcomes depending on the healthcare resource utilization. The governments should be concerned with universal health coverage in health policies for low- and middle-income countries. Investigating the major social health insurance program in Indonesia, Erlangga et al. (45) indicated that the health insurance program, subsidized by the Indonesian government, enhances the utilization of outpatient and inpatient care. In particular, the program benefits individuals who need comprehensive hospitalization and medical treatment since inpatient care is relatively expensive in healthcare in Indonesia. The process of human capital accumulation could be inefficient as the negative impact of health on economic exits in a country. The inefficiency likely derives from systemic and institutional causes in public administration and the different market structures of health services in the field of health policy services (1). In developing and emerging countries such as Myanmar, Nepal, Bangladesh, India, Pakistan, and Indonesia, inequality in health care use exists in society. The opportunity for healthy home care practices is incrased in higher socioeconomic individuals rather than lower socioeconomic individuals (46). The government should concern the policy in which lower socioeconomic individuals would benefit from health care systems. Resource allocation and governance in healthcare are vital for the government to benefit the individual's health (47). Thus, the governments should account for the problem such as corruption, bureaucracy, underinvestment, and inefficiency in health-related resources utilization.

Therefore, understanding a health policy's potential benefits and problems, with reference to the political climate, and with the uncertainty in the economic environment, can teach some interesting lessons to policymakers. Since the relationship between health and economic growth varies in different levels of health care status quo and economic development in a country, it is important to identify which area the country is in.

Western economies link with Asian countries in terms of economic development and the spread of products and jobs worldwide. Globalization has linked economies worldwide and increased the interdependence of global markets in the past three decades. From a macroeconomic perspective, international trade in globalization benefits developed countries (48). The consumption of commodities such as electrical and machinery products could not suffer from disruption in developed countries in the Western world, while a healthy and abundant workforce in major Asian countries contributes to labor-intensive manufactured exports (49). On the other hand, from a microeconomic perspective, human capital and technology are key components explaining economic growth. In major developed countries such as the United States, a highly educated workforce is an important element for a company to provide innovative products and services in technology and knowledge-intensive industries. People from Asian countries have represented the largest share of the workforce in the Silicon Valley of the United States where the world's largest high-tech companies and thousands of tech start-ups are (50). Health is an important factor in the productivity of workers, as poor health can impair performance and reduce the quality of the labor workforce (51). Thus, possessing a healthy labor force is vital for Asian and Western economies where firms and consumers gain from specialization in a global supply chain.

Previous studies have been unable to clarify the heterogeneous status linkages between health and economic growth in Asia under different health-economic conditions. The time series models used in past research could not detect the deeper connection between health and economic growth at different quantiles of the variables as conventional quantile regression is used to measure an explanatory variable's influence on the quantiles of another variable. Thus, the evidence may be inadequate for the policymakers making decisions in the aspect of health-related policy. This study used the quantile-on-quantile method, which allows heterogeneous status to be studied in light of its results. Thus, the potential connection and inadequate related researches in the health-economic nexus prompt us to contribute to this area. This study was conducted in Asian countries, and evaluation and comparison on various geographic or worldwide economies are recommended to validate the impact of health on economic growth in future research.

## Data Availability Statement

The original contributions presented in the study are included in the article/supplementary material, further inquiries can be directed to the corresponding authors.

## Author Contributions

C-FW contributed to the research topic, research model, statistical analysis, and writing. TC contributed to the research topic and research model. C-MW contributed to the data collection. T-PW contributed to the data collection. M-CL contributed to the statistical analysis and writing. S-CH contributed to the statistical analysis and writing. All authors contributed to the article and approved the submitted version.

## Conflict of Interest

The authors declare that the research was conducted in the absence of any commercial or financial relationships that could be construed as a potential conflict of interest.

## Publisher's Note

All claims expressed in this article are solely those of the authors and do not necessarily represent those of their affiliated organizations, or those of the publisher, the editors and the reviewers. Any product that may be evaluated in this article, or claim that may be made by its manufacturer, is not guaranteed or endorsed by the publisher.
